# PPARα deficiency causes skin dysbiosis and triggers innate immunity in keratinocytes

**DOI:** 10.1038/s41419-026-08640-1

**Published:** 2026-04-14

**Authors:** Stefan Blunder, Deborah Minzaghi, Petra Pavel, Michael Berktold, Martin Hermann, Christian Ploner, Cristina Schöpf, Florentine Marx, Sandrine Dubrac

**Affiliations:** 1 Department of Dermatology, Venereology and Allergology, Medical University of Innsbruck, Innsbruck, Austria; 2 Department of Dermatology, Perelman School of Medicine, University of Pennsylvania, Philadelphia, USA; 3 Institute of Hygiene and Medical Microbiology, Medical University of Innsbruck, Innsbruck, Austria; 4 Department of Anesthesiology and Intensive Care Medicine, Medical University of Innsbruck, Innsbruck, Austria; 5 Department of Plastic, Reconstructive and Aesthetic Surgery, Medical University of Innsbruck, Innsbruck, Austria; 6 Biocenter, Institute of Molecular Biology, Medical University of Innsbruck, Innsbruck, Austria

**Keywords:** Immunology, Innate immunity

## Abstract

Peroxisome proliferator-activated receptor-α (PPARα) is a transcription factor expressed in the skin, where its physiological role remains largely unknown. In this work, we found decreased bacterial density and dysbiosis associated with reduced amounts of *Staphylococcus* in the skin of PPARα-deficient mice compared to littermate controls. The NOD2 pathway was enhanced, as shown by up-regulation of both NOD2 and its downstream targets, including the antimicrobial peptides mBD3 and mBD4. Moreover, the Th17 immune response was enhanced in keratinocytes and epidermal dendritic T cells. Topical treatment of PPARα-deficient mice with an antibacterial solution did not normalize the upregulation of the innate immunity in keratinocytes. However, the up-regulation of FLG and excessive oxidative stress detected in the epidermis of PPARα-deficient mice were markedly inhibited by topical antibacterial treatment. Thus, our study highlights the overarching role of PPARα in shaping skin immune system and microbiota, with the latter largely controlling FLG expression and inducing oxidative stress in keratinocytes.

## Introduction

Peroxisome proliferator-activated receptor α (PPARα) is a nuclear hormone receptor that heterodimerizes with the retinoic X receptor to function as a transcription factor regulating glucose, amino acid and lipid metabolism [1]. Long-chain fatty acids, including various eicosanoids, oxidized fatty acids, and phospholipid derivatives, are natural PPARα ligands and they have been found in the epidermis [2, 3]. In epidermis, PPARα has been found in melanocytes, Langerhans cells (LCs) and suprabasal keratinocytes [1]. PPARα is also expressed in cutaneous immune cells, such as dendritic cells, lymphocytes, macrophages and mast cells [4, 5]. In the skin, PPARα activation with pharmacological doses of ligands (e.g., WY14643) produces anti-inflammatory effects and promotes epidermal differentiation [6]. PPARα has been shown to exert its effects via modulation of NF-κB and AP-1. However, some of the effects of PPARα ligands are mediated via receptor-independent mechanisms [7].

PPARα expression is reduced in skin lesions of patients with atopic dermatitis or psoriasis, whereas PPARα deficiency is associated with exacerbated skin inflammation in various mouse models [3, 8–11]. In addition, PPARα deficiency was shown to alter lipid metabolism and differentiation processes in mouse keratinocytes [6]. The primary role of PPARα is the regulation of fatty acid oxidation in both mitochondria and peroxisomes. However, the epidermis is characterized by high anabolic rates of fatty acids, as opposed to fatty acid catabolism. Thus, despite significant advancements in dissecting the role of PPARα in the skin, it is likely that many PPARα functions have not yet been discovered. The skin immune system shapes the skin microbiota and PPARα is an important transcription factor that controls the function of immune cells and keratinocytes. Consistent with this, studies have shown that PPARα deficiency can lead to intestinal dysbiosis, but can also protect mice against pulmonary bacterial superinfection with *Staphylococcus aureus* following primary influenza virus infection by promoting lung inflammation and inhibiting necroptosis [12, 13]. The role of PPARα in shaping the landscape of skin microbiota has not yet been investigated. Therefore, the goal of this work was to investigate yet-unexplored pathways controlled by PPARα in the epidermis, including effects on the skin microbiota.

## Materials and methods

All materials are listed in Supplementary Table S3.

### Ethics approval and consent to participate

- All methods were performed in accordance with the relevant guidelines and regulations.
- Animal experiments were conducted under a protocol approved by the Institutional Animal Care Committee (BMWFW.66-011/0004-WF/V/3b/2017).

### Mice

PPARα-deficient and littermate control mice were bred on a C57BL6/129S background as described previously [10]. Briefly, to generate PPARα-deficient mice lacking exon 8 of the *Ppara* gene—which together with exon 7 encodes the ligand-binding domain of the protein—a 1.14 kb neomycin resistance gene was inserted in the opposite transcriptional direction to disrupt exon 8 [14]. Three to five-month-old male (29 g ± 1) and female (25 g ± 1) mice were used for all experiments. Mouse whole body skin including ears has been used for experiments. Mice were topically treated on their whole body surface with an antimicrobial solution (AMS) for 5 days twice a day.

### Analysis of skin bacteria

Mice were housed in individual cages for 5 weeks prior to skin microbiota analyses. Colony-forming units (CFU) were determined by homogenizing an 8-mm mouse skin punch biopsy in phosphate-buffered saline (PBS) and further culturing the homogenates onto agar media at 37 °C overnight. Alternatively, skin swabs were taken from the whole mouse body surface, collected in sterile tubes, snap frozen in liquid nitrogen and stored at -80 °C until analysis. DNA was extracted with an Ultraclean swab DNA Isolation Kit according to manufacturer´s instructions (MoBio Laboratories, Carlsbad, CA). The 16S universal Eubacterial primers 515F GTGCCAGCMGCCGCGGTAA and 806R GGACTACHVGGGTWTCTAAT were utilized to evaluate the microbial ecology of each sample on an Illumina MiSeq instrument using bTEFAP® methodology provided by a commercial DNA analysis service (MR DNA, Shallowater, TX). A single-step 30-cycle PCR using HotStarTaq Plus Master Mix Kit (Qiagen, Valencia, CA) was used under the following conditions: 94 °C for 3 min, followed by 28 cycles of 94 °C for 30 s; 53 °C for 40 s and 72 °C for 1 min; after which a final elongation step at 72 °C for 5 min was performed. Following PCR, all amplicon products from different samples were mixed in equal concentrations and purified using Agencourt Ampure beads (Agencourt Bioscience Corporation, MA). Samples were sequenced utilizing Illumina MiSeq chemistry following the manufacturer’s protocols. The Q25 sequence data derived from the sequencing process was processed using a proprietary analysis pipeline (www.mrdnalab.com, MR DNA, Shallowater, TX). Sequences were depleted of barcodes and primers, and sequences <200 bp, sequences with ambiguous base calls, and sequences with homopolymer runs >6 bp were removed. Sequences were then denoised and chimeras removed. Operational taxonomic units (OTUs) were defined after removal of singleton sequences, clustering at 3% divergence (97% similarity) [15–18]. OTUs were then taxonomically classified using BLASTn against a curated GreenGenes/RDP/NCBI-derived database [19]. Alternatively, the presence of *Staphylococcus* (*S*.) *lentus* on mouse skin was assessed by PCR and qPCR for *S. aureus*, *S. epidermidis*, and 16SrRNA. Relative abundance of *S. aureus* and *S. epidermis* was calculated against 16S (threshold cycle (CT) bacteria/CT 16S).

### Skin cell analysis

Whole-skin digestion was achieved by digestion with Liberate^TM^ in the presence of DNAse [20]. Then, single-cell suspensions were stained using directly labeled primary monoclonal antibodies. Skin cells including neutrophils, M1/M2 macrophages, eosinophils, and LCs were identified as depicted in Supplementary Table S1. Whole skin suspension was alternatively re-stimulated in vitro with phorbol 12-myristate 13-acetate and ionomycin in the presence of Golgi plug for 4 h [10]. Then, the cells were directly labeled with primary monoclonal antibodies. Intracellular staining for IFN-γ, IL-17A, and IL-13 was carried out after fixation and permeabilization of cells with a cell permeabilization kit. Cells were analyzed by flow cytometry after exclusion of dead cells by live-dead or 7AAD staining. Gating strategy is reported in Supplementary Fig. S3D. Mast cells were stained with Giemsa and counted with a grid (10 fields per sample).

### Quantification of gene expression

Total RNA was extracted from mouse epidermis using TRIZOL reagent (Life Technologies, Carlsbad, CA), genomic DNA was removed from the RNA preparation (Ambion, Carlsbad, CA) according to the manufacturer’s protocol. RNA quantity was determined with a Nanodrop (Waltham, MA) and its integrity was evaluated with a fragment analyzer (Advanced Analytical, Heidelberg, Germany). Thereafter, reverse transcription was carried out and quantitative qPCR analysis was performed by real-time PCR (real-time PCR detection system CFX96; Bio-Rad, Vienna, Austria) using a Brilliant III Ultra-Fast Quantitative PCR Kit from Agilent Technologies (Vienna, Austria) [9, 21]. *Tbp* and *Ppia* were used as the housekeeping genes.

### Morphological analysis and immunofluorescence

Skin biopsies were fixed in 4% formaldehyde, paraffin-embedded, and 6-µm sections were stained with hematoxylin & eosin. Sections were analyzed using an Olympus BH-2 light microscope (Olympus, Shinjuku, Japan) equipped with a ProgRes C10plus camera (Jenoptik, Jena, Germany), and ProgResCapturePro 2.8.8 image analysis software (Jenoptik, Jena, Germany). Paraffin-embedded skin biopsies were deparaffinized and subjected to heat-mediated antigen retrieval by using sodium-citrate buffer (10 mM sodium citrate, 0.05% Tween, pH 6.0). The sections were then blocked with 10% goat serum for 60 min at room temperature and subsequently incubated with primary antibodies (Supplementary Table S3) in 2% goat serum overnight at 4 °C or 1 h at room temperature. Sections were then stained with secondary antibodies, i.e., AlexaFluor488 goat anti-rabbit IgG or AlexaFluor594 goat anti-mouse IgG (Thermo Fisher Scientific, Waltham, MA) for 45 min at room temperature in the dark. Nuclei were stained with 4′,6-diamidino-2-phenylindole (DAPI). Thereafter, sections were mounted in VectaShield (antifade mounting medium, Vector Laboratories, Burlingame, CA) and confocal images acquired using a LEICA DMLS Microscope (Leica Mikroskopie und Systeme, Wetzlar) [9, 22]. Intensity of immunofluorescence staining was measured with ImageJ and reported in the Supplementary Fig. S10A.

### Western blot analysis

Epidermis was lysed in modified RIPA buffer (10 mM Tris-HCl, 150 mM NaCl, 1 mM EDTA, 1% Triton X-100, 1% sodium deoxycholate, 0.1% SDS, pH 7.4) in the presence of protease inhibitor (Pierce, Rockford, IL). Endogenous proteins (reducing conditions, DTT) were detected with primary and secondary antibodies as listed in Supplementary Table S3. Blots were then scanned with a LI-COR Biosciences analyzer (Lincoln, NE) and band intensity was analyzed with Image Studio v3.1 software from LI-COR Biosciences. A pool of 5 mice was used. VDAC was used as loading control. Full Western blots are provided in Supplementary Fig. S11.

### H_2_O_2_ measurements

Mouse epidermis was incubated in keratinocyte culture medium (CnT-07, CellnTec, Bern, Switzerland) for 24 h. H_2_O_2_ was quantified in medium using an Amplex® Red Hydrogen Peroxide/Peroxidase Assay Kit (Invitrogen, Carlsbad, CA) using 50 µl samples as described by the manufacturer. Proteins were prepared from the epidermis for normalization.

### Transmission electron microscopy

Epidermis was cut into 2 mm × 2 mm pieces, fixed in modified Karnovsky’s solution, and then rinsed twice in 0.1 M cacodylate buffer. Thereafter, samples were processed using osmium tetroxide (OsO4) post-fixation staining and visualized with a Zeiss 10A (Carl Zeiss, Jena, Germany) or Hitachi HT 7700 (Tokyo, Japan) electron microscope [9, 21, 23].

### Enzymatic activity of complex I

Mitochondrial fractions were prepared from fresh mouse epidermis by sequential centrifugation in Tris-sucrose buffer containing bovine serum albumin. Briefly, complex I activity was measured using a dichlorophenolindophenol (DCPIP)-coupled method, which is based on the reduction of DCPIP by electrons from decylubiquinol, reduced by complex I after NADH oxidation [9, 21].

### Epidermal glycerol analysis

Epidermal glycerol was quantified by using a kit from Sigma-Aldrich (St. Louis, MO) according to the supplier´s instructions.

### Statistical analysis

Statistical analyses were performed using GraphPad Prism software version 10. Results are expressed as mean ± SEM and analyzed for statistical significance by using an unpaired, two tailed Student’s *t* test. Normal distribution was assumed and the variance was similar between groups. Different sets of mice have been used to carry out all experiments, *n* = 3–10.

## Results

### PPARα deficiency reduces skin bacterial colonization in the mouse

**Fig. 1:**
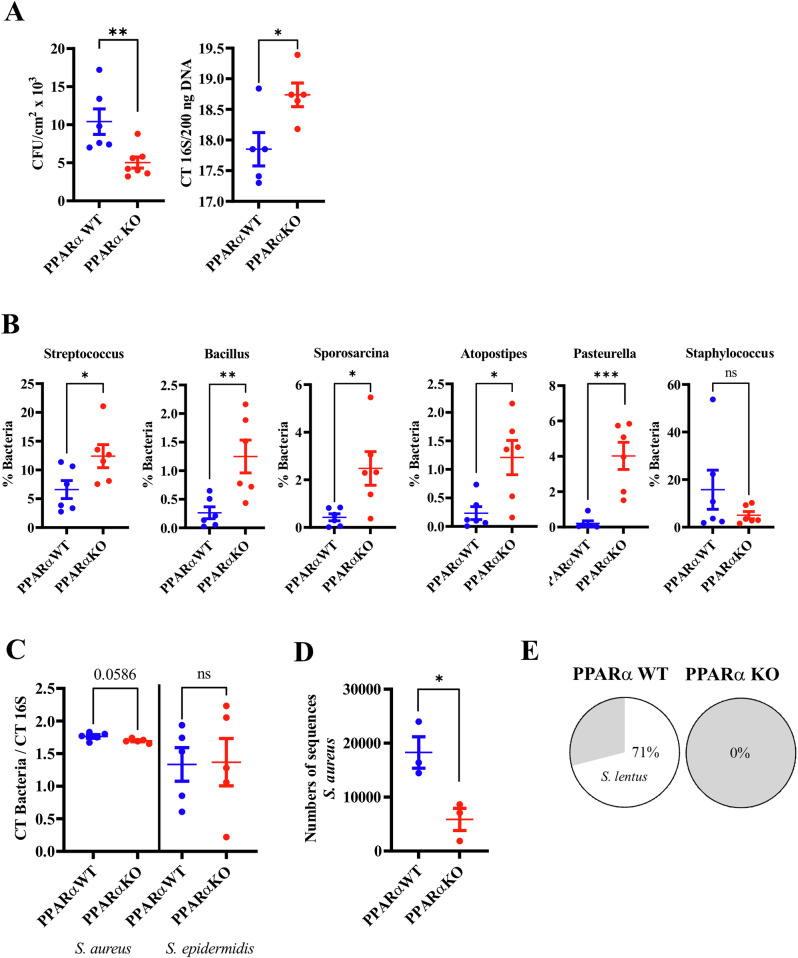
PPARα deficiency reduces skin bacterial colonization. **A** Colony-forming units (CFUs) and qPCR showing relative amounts of bacteria on mouse skin. **B** Proportions of bacterial species on mouse skin. **C** qPCR showing relative amounts of *S. aureus* and *S. epidermidis* on mouse skin. **D** Numbers of sequences for *S. aureus* calculated with the Illumina MiSeq analysis. **E** Percentage of mice positive for *S. lentu*s in the skin. Data were analyzed with an unpaired Student’s *t* test, *n* = 3–7. *, *p* < 0.05; **, *p* < 0.01.

To unravel new pathways controlled by PPARα in the epidermis, we utilized PPARα-deficient mice and their littermate controls. We first investigated whether PPARα deficiency might control skin bacterial colonization. We found reduced bacterial density in PPARα-deficient mice when compared to littermate controls (Fig. 1A, left panel), which was confirmed by qPCR (Fig. 1A, right panel), demonstrating that PPARα deficiency hinders bacterial growth in the skin. Next, we studied the bacterial composition of mouse skin. The proportions of *Sporosarcina*, *Atopostipes*, *Bacillus* and *Streptococcus*—all Gram-positive Firmicutes—and of *Pasteurella*, a Gram- negative Proteobacteria, were increased in the skin of PPARα-deficient mice when compared to littermate controls (Fig. 1B, Supplementary Table S2). In contrast, the relative proportion of *Staphylococcus* was reduced, although the difference did not reach significance (Fig. 1B). To refine our understanding of the effects of PPARα deficiency on these bacterial populations, we carried out qPCR analyses. The results showed a borderline reduction of the relative quantity of *S. aureus*, in contrast to that of *S. epidermidis* where no reduction was evident (Fig. 1C). Reduced *S. aureus* on PPARα-deficient mouse skin was confirmed by the numbers of sequences calculated with the Illumina MiSeq analysis (Fig. 1D). Moreover, complementary analysis of mouse skin bacteria revealed the presence of *S. lentus* in the skin of 5 out of 7 PPARα wild-type mice, whereas this bacterium was never detected on the skin of PPARα-deficient mice (*n* = 4) (Fig. 1E). *S. lentus* is one of the most abundant staphylococcal species on mouse skin, and its reduction likely contributes to the overall reduced proportion of *Staphylococcus* in PPARα-deficient mice [24]. Thus, taken together, these data show that PPARα deficiency decreases bacterial colonization, induces dysbiosis and alters the proportions of *Staphylococcus* species in mouse skin.

### PPARα deficiency augments β-defensins and triggers the NOD2 pathway in the epidermis

**Fig. 2:**
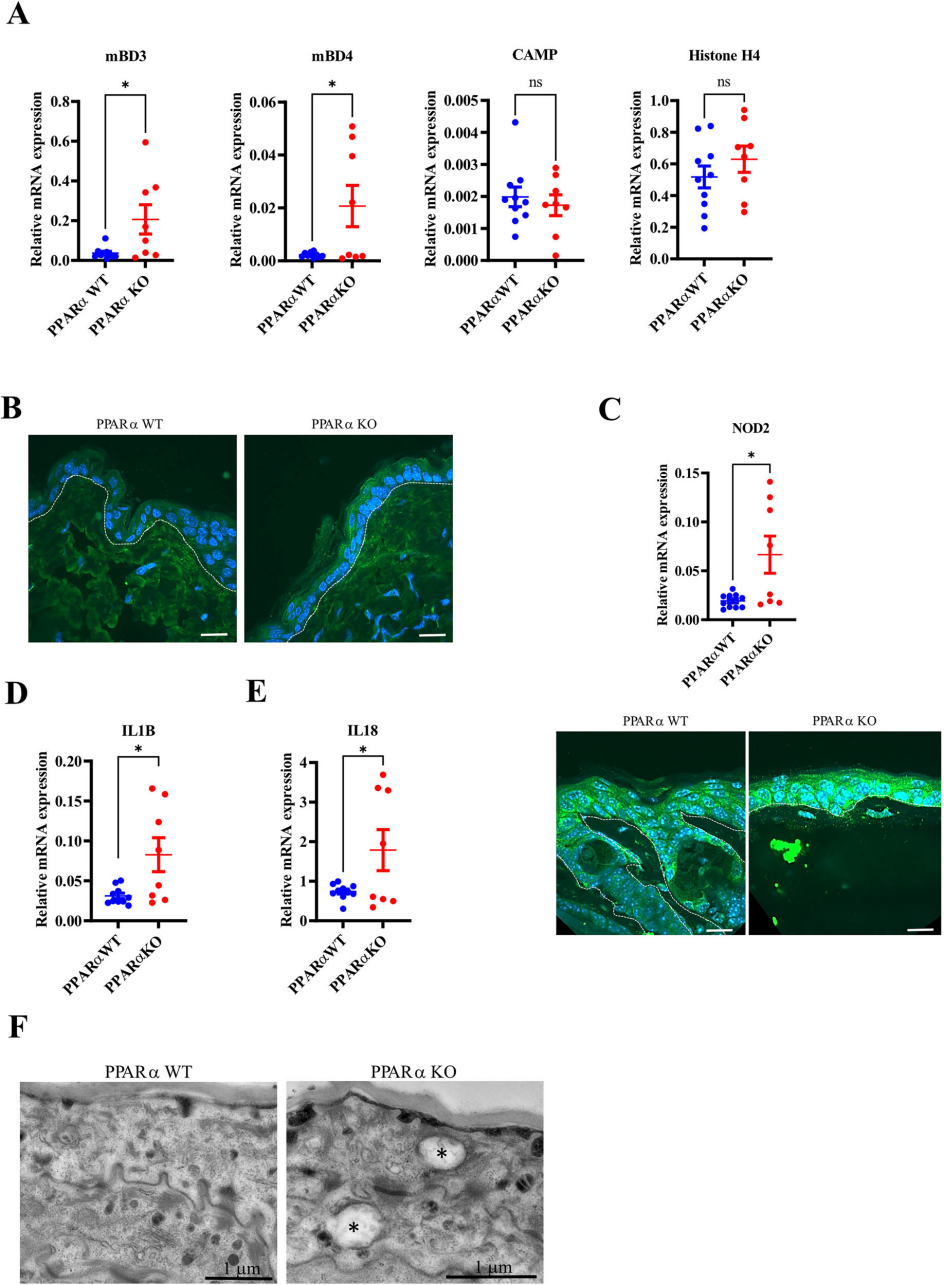
PPARα deficiency triggers β-defensins and the NOD2 pathway in the epidermis. **A** Relative mRNA levels of various antimicrobial peptides in mouse epidermis. **B** Immunostaining of mBD3 in mouse skin; Dashed line: epidermis/dermis border zone. **C** Relative mRNA levels and representative picture of immunostaining and Western blot analysis of NOD2. TBP served as loading control. Relative mRNA levels of *Il1B* (**D**) and *Il18* (**E**) in mouse epidermis. **F** Transmission electron microscopy of mouse epidermis (*n* = 3) showing autophagosomes (*) in the stratum granulosum of PPARα-deficient mice. Scale bar = 1 μm. Data were analyzed with an unpaired Student’s *t* test, *n* = 8–10. *, *p* < 0.05.

Alterations in the skin microbiota might result from modification of several parameters, including the integrity of the stratum corneum, skin surface acidity, and the production of defensins [25]. We first investigated the expression of key players of epidermal innate immunity. We found that the expression of *Defb3* and *Defb4*, encoding for the antimicrobial β-defensins mBD3 and mBD4 respectively, was increased in the epidermis of PPARα-deficient mice when compared to littermate controls, in contrast to *Camp* and *Hist4h4*, which code for mouse cathelicidin and histone H4, respectively (Fig. 2A). Moreover, immunostaining for mBD3 was increased in the epidermis of PPARα-deficient mice (Fig. 2B). We also found increased *Il1b* (Fig. 2D) and *Il18* (Fig. 2E) mRNA levels in the epidermis of PPARα-deficient mice when compared to littermate controls. Because NOD2 is an intracellular pattern recognition receptor expressed in keratinocytes that controls the expression of defensins and inflammasome-related cytokines such as IL-1β and IL-18 [26–29], these results strongly suggested the implication of NOD2. In line with this, we found enhanced expression of *Nod2* in the epidermis of PPARα-deficient mice when compared to littermate controls (Fig. 2C). TEWL and surface skin pH were not altered in PPARα-deficient mice compared to littermate controls (Supplementary Fig. S1A, B). Transmission electron microscopy (TEM) confirmed no alteration of the lamellar body secretory system but did reveal autophagosomes only in PPARα-deficient mouse epidermis (Fig. 2F). Thus, our results show that PPARα deficiency triggers β-defensins and inflammasome-related cytokines, potentially via activation of the NOD2 pathway.

### PPARα deficiency triggers subclinical signs of epidermal Th17 inflammation

**Fig. 3:**
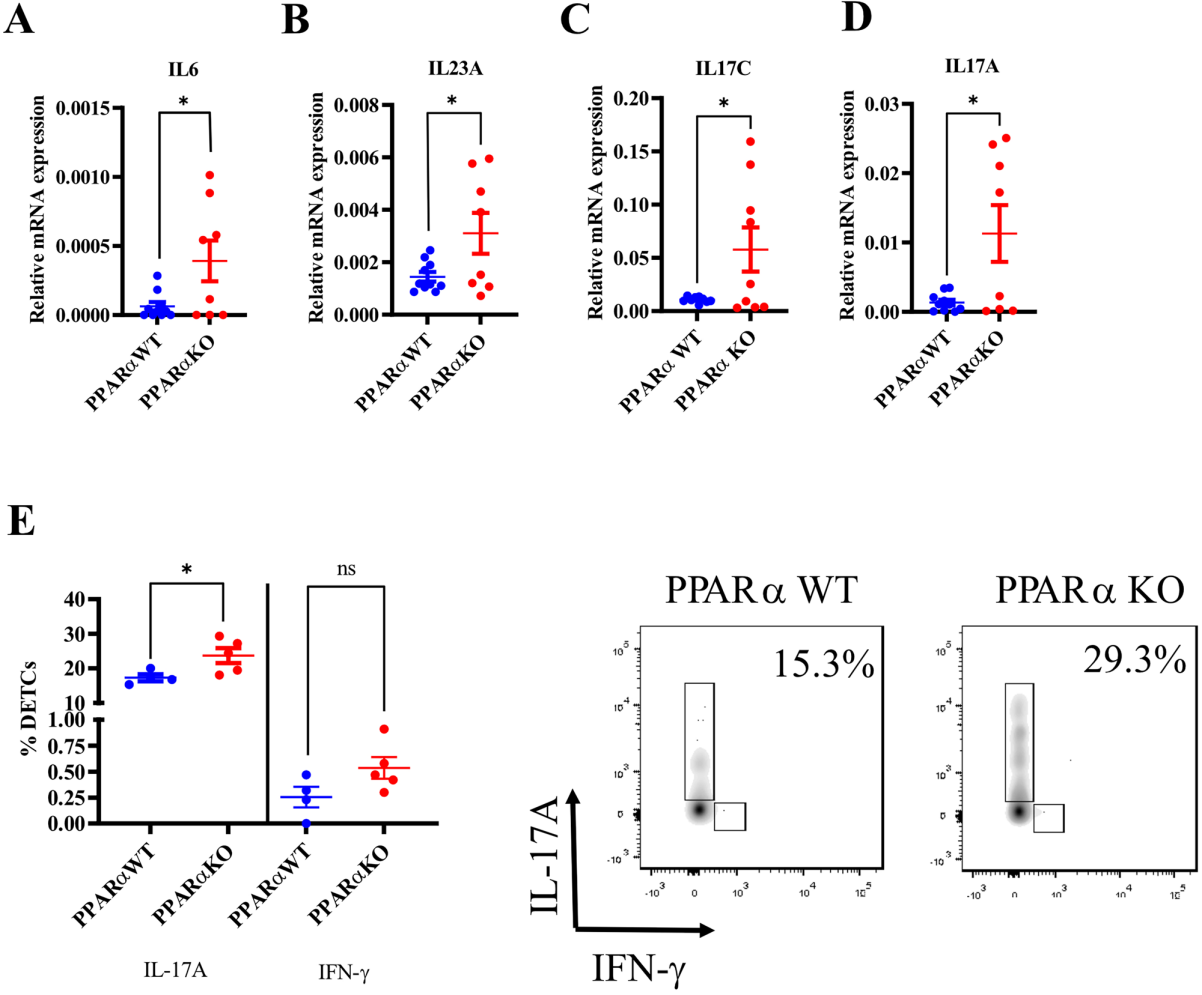
PPARα deficiency triggers subclinical signs of epidermal Th17 inflammation. Relative mRNA levels of *Il6* (**A**), *Il23a* (**B**), *Il17c* (**C**) and *Il17a* (**D**) in mouse epidermis. **E** Percentages of DETCs in mouse epidermis producing IL-17A or IFN-γ. Numbers in FACS plots show percentages of IL-17A^+^DETCs. Data were analyzed with an unpaired Student’s *t* test, *n* = 8-10. *, *p* < 0.05.

Up-regulation of β-defensins is not the only mechanism able to eliminate bacteria such as *Staphylococcus* in the skin [30, 31]. Increased Th17 immunity has been shown to exert anti-staphylococcal effects [32, 33]. The expression of *Il6* and *Il23a* as well as the expression of *Il17c* and *Il17a* was enhanced in the epidermis of PPARα-deficient mice when compared to littermate controls (Fig. 3A-D), unlike the expression of *Il17e/Il25*, which was unchanged (Supplementary Fig. S2A). A Th17-enriched microenvironment induces CXCL1, S100A8 and VEGF [34]. In line with this, we observed increased *Cxcl1*, *S100a8* and *Vegfa* mRNA levels in the epidermis of PPARα-deficient mice (Supplementary Fig. S2B-D), consistent with increased release of Th17 cytokines (Fig. 3). The expression of alarmins such as *Il33* remained unchanged, similar to the expression of *Il1a*, *Tgfb* and *Tnfa* (Supplementary Fig. S2E-I). We did not detect *Il22* in mouse epidermis. Type 2 DCs (cDC2s) and LCs bridge the innate and the adaptive immune system and prime Th17 cells [32, 33, 35]. We found increased percentages of cDC2s and of LCs but not of cDC1s in the skin of PPARα-deficient mice (Supplementary Fig. S3A, B). Next, we studied the DETC population in mouse epidermis. We did not find any significant differences in the proportions of DETCs in the epidermis of PPARα-deficient mice when compared to controls (Supplementary Fig. S3C). Nevertheless, we found higher production of IL-17A in restimulated DETCs of PPARα-deficient mice (Fig. 3E, Supplementary Fig. S3D). Th1 and Th2 responses were not altered (Supplementary Fig. S3E). LCs have previously shown to promote expansion of regulatory T cells, thereby contributing to skin tolerance to commensal bacteria [36, 37]. Although PPARα-deficient epidermis contains more LCs compared to wild-type epidermis, the expression of *Foxp3* and *Il10* was similar in both mouse groups (data not shown) as reported earlier [10]. Thus, PPARα deficiency augments LCs and cDC2s in mouse skin and triggers subclinical signs of a Th17 immune response in mouse keratinocytes and DETCs, which could contribute to the elimination of bacteria (Fig. 1A) and especially of *Staphylococcus* (Fig. 1C, D).

### Reduction of skin bacteria in PPARα-deficient mouse skin does not result from increased recruitment of innate immune cells to the skin

Innate cells such as neutrophils and macrophages are important cells able to control the skin microbiota [38, 39]. We found similar proportions of neutrophils, macrophages (M1 and M2), and mast cells in the skin of PPARα-deficient and littermate control mice (Supplementary Fig. S4A-B, E), despite an increased expression of *Gmcsf* by keratinocytes, which is involved in the chemoattraction and activation of neutrophils and macrophages [40] (Supplementary Fig. S4C). Moreover, expression of the activation marker CD86 at the cell surface of M1/M2 macrophages remained unchanged, although CD11b on neutrophils was reduced in PPARα-deficient mice when compared to littermate controls (Supplementary Fig. S4D). Eosinophils were largely absent from mouse skin (data not shown) and mast cell numbers were unchanged (Supplementary Fig. S4E). Thus, the reduction of bacteria in the skin of PPARα-deficient mice is not due to increased recruitment or activation of innate cells.

### Oxidative stress is triggered in PPARα-deficient epidermis

**Fig. 4:**
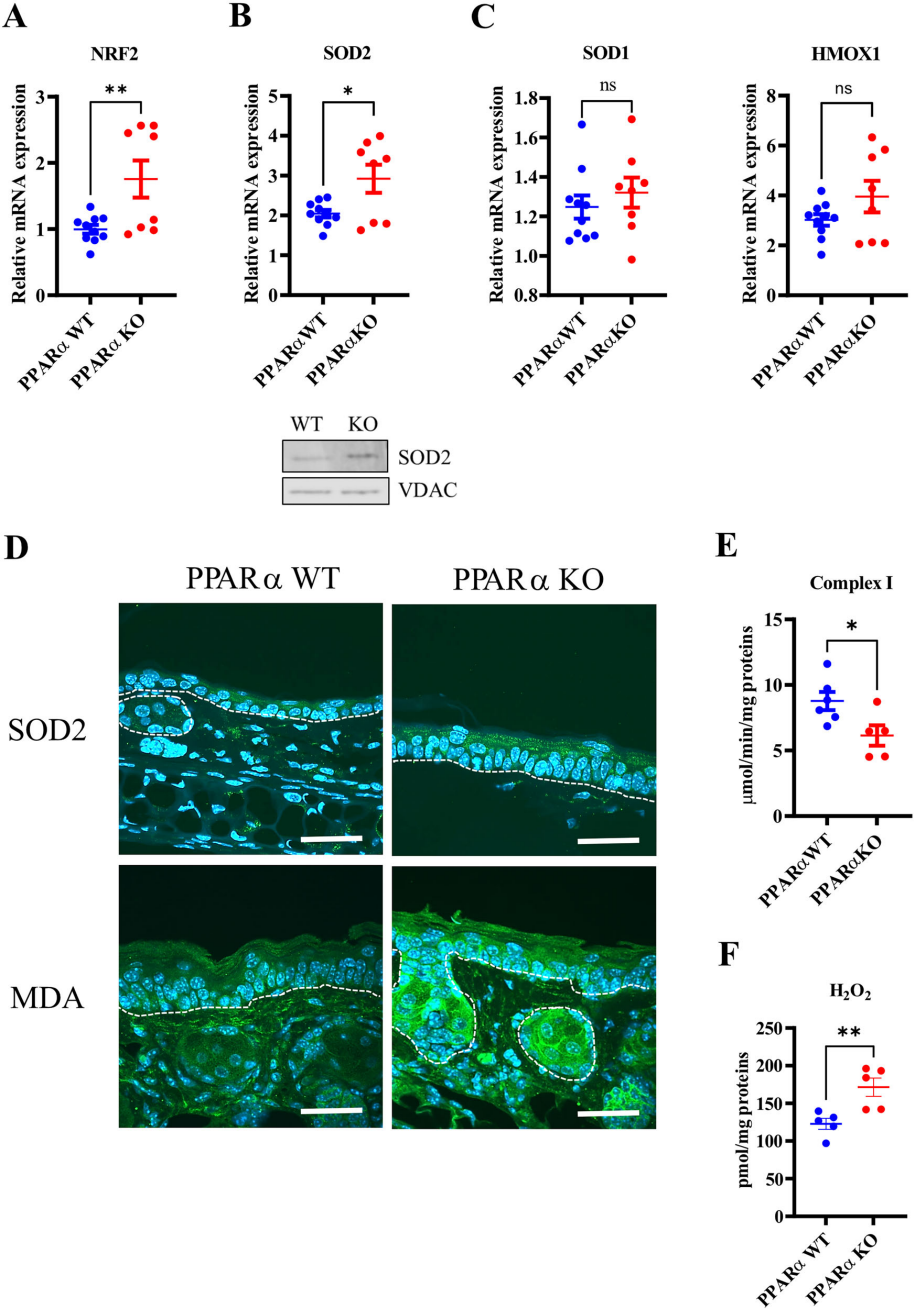
Oxidative stress is triggered in PPARα-deficient epidermis. Relative mRNA levels of *Nrf2* (**A**), (**B**) *Sod2*, (**C**) *Sod1* and *Hmox1* in mouse epidermis. Western blot analysis showing NRF2 (**A**) with TBP as loading control and SOD2 (**B**) with VDAC as loading control. **D** Immunostaining of SOD2 and MDA in mouse epidermis. **E** Specific activity of complex I in mouse epidermis. **F** Levels of H_2_O_2_ in mouse epidermis. Data were analyzed with an unpaired Student’s *t* test, *n* = 5–10. *, *p* < 0.05; **, *p* < 0.01.

Skin dysbiosis, especially enrichment in Firmicutes such as *Streptococcus*, can trigger oxidative and mitochondrial stress in host cells, including keratinocytes [41–43]. Thus, we hypothesized that skin dysbiosis in PPARα-deficient mice induces oxidative stress in keratinocytes. We found that PPARα-deficient mice exhibit increased expression of *Nrf2* as well as of the NRF2 mitochondrial target gene *Sod2* at both the mRNA and protein levels (Fig. 4A, B). The expression of non-mitochondrial NRF2 target genes such as *Sod1* and *Hmox1* remained unchanged (Fig. 4C). Moreover, we found increased immunostaining of malondialdehyde (MDA) in the epidermis of PPARα-deficient mice (Fig. 4D), demonstrating increased amounts of peroxidized lipids. Furthermore, basal keratinocytes expressing the DNA damage marker γH2AX were present in the epidermis of PPARα-deficient mice but absent in the epidermis of littermate controls (Supplementary Fig. S5A). Mitochondrial enzymatic activity, especially of complex I, is the main source of ROS in cells. The activity of complex I was reduced in the epidermis of PPARα-deficient mice (Fig. 4E), in the absence of variation of the quantity and distribution of mitochondria (Supplementary Fig. S5B). In line with these results, the production of H_2_O_2_ in the epidermis of PPARα–deficient mice was increased when compared to control mice (Fig. 4F). Thus, these data reveal oxidative and mitochondrial stress in keratinocytes of PPARα-deficient mice.

### AQP3 is increased in the epidermis of PPARα-deficient mice

Aquaporin-3 (AQP3) is a water channel permeable to water, glycerol and hydrogen peroxide in keratinocytes [44, 45]. We found upregulation of *Aqp3* mRNA (Supplementary Fig. 6A) and protein (Supplementary Fig. 6B) levels in the epidermis of PPARα-deficient mice when compared to littermate controls. The epidermal content of glycerol was similar in the epidermis of PPARα-deficient mice and littermate controls (Supplementary Fig. S6C). Thus, because oxidative stress is increased in PPARα-deficient keratinocytes (Fig. 4, Supplementary Fig. S5), up-regulation of AQP3 in the epidermis of PPARα-deficient mice is likely involved in hydrogen peroxide trafficking (Fig. 4F) [45].

### Alterations of epidermal homeostasis in PPARα-deficient epidermis

**Fig. 5:**
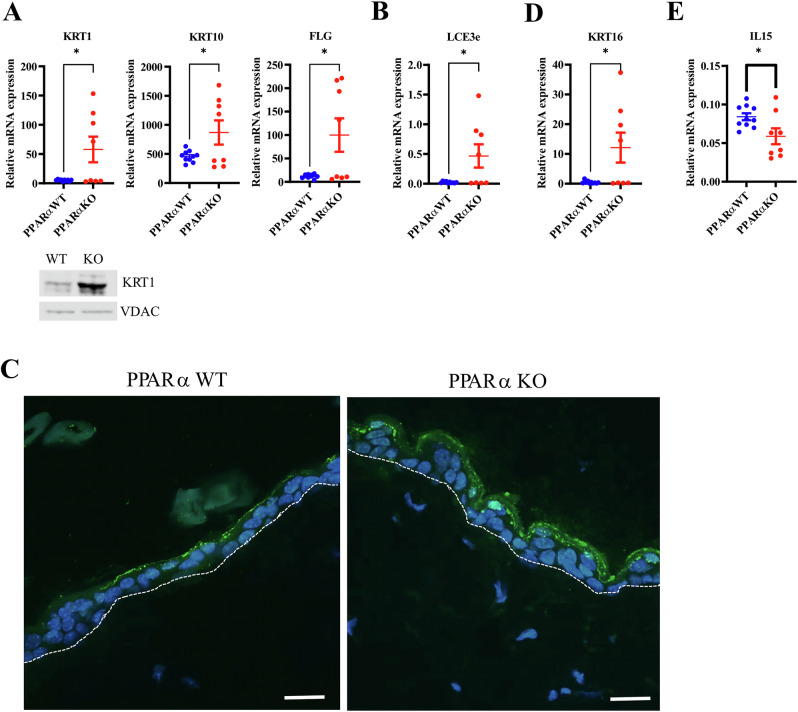
PPARα deficiency alters epidermal homeostasis. Relative mRNA levels of *Krt1, Krt10, Flg* and Western blot analysis showing KRT1 and VDAC as loading control (**A**) and *Lce3e* (**B**). **C** Immunostaining of (pro)FLG in mouse epidermis. Relative mRNA levels of *Krt16* (**D**) and *Il15* (**E**). Data were analyzed with an unpaired Student’s *t* test, *n* = 8–10. *, *p* < 0.05.

We next assessed the expression of various proliferation and differentiation markers in mouse epidermis. We found increased expression of early and late differentiation markers, namely *Krt1, Krt10, Flg*, and *Lce3e* (Fig. 5A, B). Increased amounts of differentiation markers in the epidermis of PPARα-deficient mice were confirmed at protein level for KRT1 (Fig. 5A). Moreover, the number of Ki67^+^ keratinocytes was increased in the epidermis of PPARα-deficient mice (Supplementary Fig. S9B), suggesting altered epidermal homeostasis. In line with this, *Krt16* was also up-regulated in the epidermis of PPARα-deficient mice (Fig. 5D). Moreover, we found reduced expression of *Il15* (Fig. 5E), a cytokine expressed by keratinocytes that interferes with epidermal differentiation processes [46]. To verify whether these changes translated into macroscopic changes in epidermal architecture, we carried out hematoxylin and eosin (H&E) staining on mouse skin. We did not find gross changes as reported earlier [1], although a consistent mild hyperkeratosis was noted in PPARα-deficient mice (Supplementary Fig. S7A). This was not due to an altered desquamation process, as the expression of both *Spink5* and *Klk5* was not altered (Supplementary Fig. S7B). All together, these results show that PPARα deficiency leads to higher keratinocyte turnover.

### Epidermal lipid metabolism is not affected by PPARα deficiency

In the liver, PPARα regulates glucose and lipid metabolism [47]. Expression of canonical PPARα target genes involved in mitochondrial and peroxisomal lipid metabolism (*Echs1*, *Cpt1a*, *Hadhb*, *Acadvl*, *Acox1*, *Hsd17b4*) was not changed in the epidermis of PPARα-deficient mice (Supplementary Fig. S8A, B). Moreover, no overexpression of *Pparg* and *Ppard* was observed (Supplementary Fig. S8C), precluding compensatory mechanisms via other PPAR isoforms. Thus, these findings show that the pattern of genes regulated by PPARα in the epidermis is different from that in other organs. Moreover, these results suggest that the role of PPARα in mouse epidermis is not primarily in lipid metabolism.

### Topical treatments with an antimicrobial solution highlight major effects of PPARα deficiency in epidermal innate immunity

**Fig. 6:**
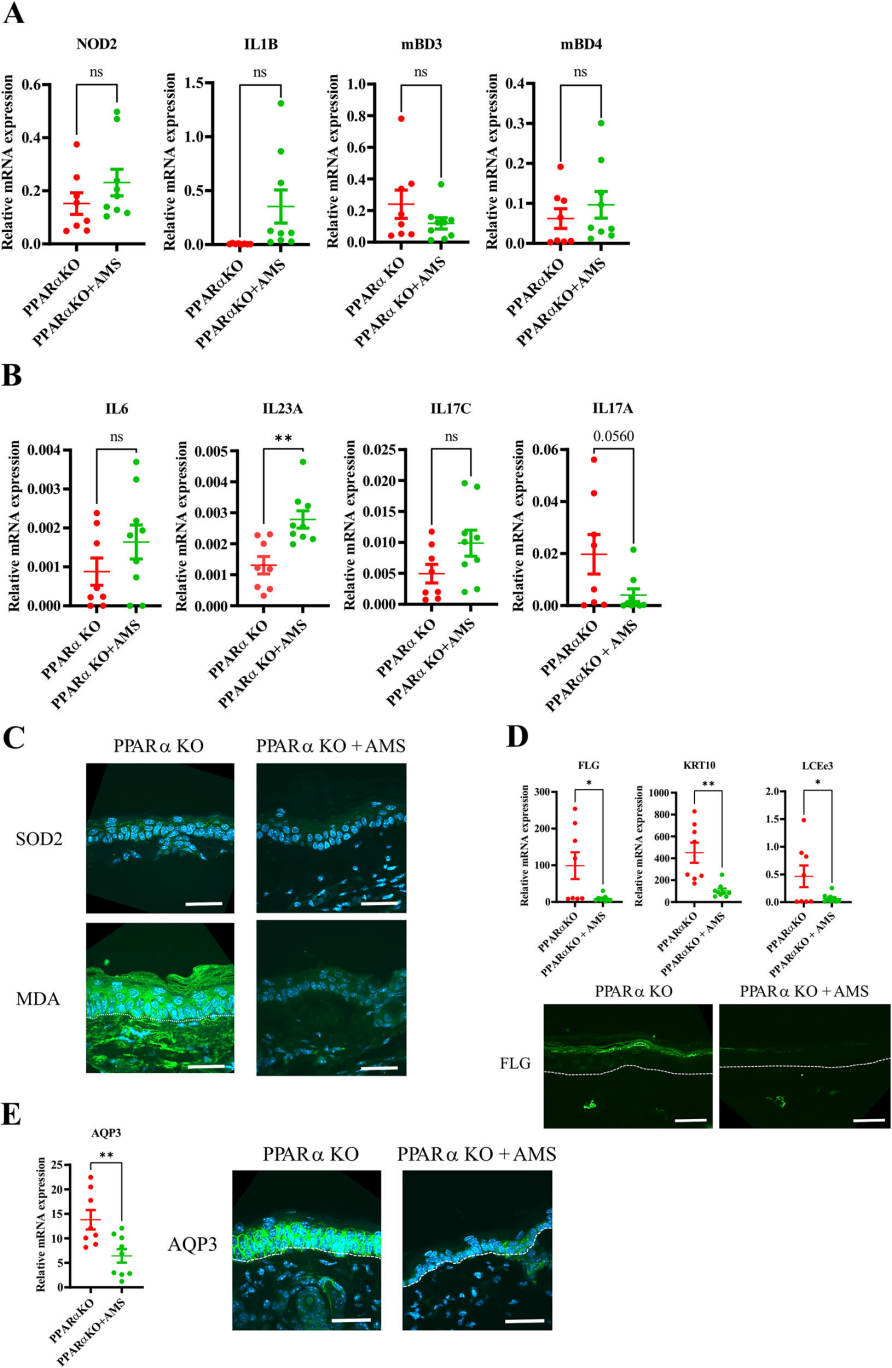
Topical treatments with an antimicrobial solution highlight major effects of PPARα deficiency in epidermal innate immunity. Mice were topically treated with an antimicrobial solution as described in Materials and Methods. **A** Relative mRNA levels of *Nod2*, *Il1b, mBD3*, and *mBD4*. **B** Relative mRNA levels of *Il6*, *Il23a*, *Il17c*, and *Il17a*. **C** Immunostaining of SOD2 and MDA. **D** Relative mRNA levels of *Krt10*, *Flg* and *Lce3e* and immunostaining of FLG. **E** Relative mRNA level and immunostaining of AQP3 in mouse epidermis. Data were analyzed with an unpaired Student’s *t* test, *n* = 8–9. *, *p* < 0.05; **, *p* < 0.01.

In order to distinguish effects owing to PPARα deficiency from those due to bacterial dysbiosis, we topically treated PPARα-deficient mice with an antimicrobial solution (AMS). Figure 6A and S9A show similar levels of expression of *Defb3*, *Defb4*, *S100a8*, *Nod2* and *Il1b* in both AMS-treated and untreated PPARα-deficient mice, demonstrating that PPARα deficiency, per se, triggers epidermal innate immunity via upregulation of the Nod2 pathway. Topical treatment with AMS did not dampen the Th17 immune response in keratinocytes, as opposed to its effect on DETCs (Fig. 6B). This is in line with previous work showing that, in a sterile environment, PPARα deficiency does not trigger IL-17A in mouse and human T cells [48, 49]. In contrast, SOD2 and MDA levels were reduced in the epidermis of PPARα-deficient mice treated with AMS when compared to untreated PPARα-deficient mice (Fig. 6C), pointing to a primary role of skin dysbiosis in the oxidative stress evidenced in PPARα-deficient epidermis. In line with this, we observed normalization of AQP3 at the mRNA and protein levels in the epidermis of PPARα-deficient mice after topical treatment with AMS (Fig. 6E). We next studied the effects of AMS on keratinocyte differentiation. We found that mRNA levels of *Krt10*, *Flg* and *Lce3e* were normalized by AMS treatment. In line with these data, FLG protein levels were also restored following AMS treatment. (Fig. 6D). In contrast to that *Krt1* and *Krt16 mRNA levels* and the numbers of Ki67^+^ keratinocytes were not altered upon AMS treatment (Supplementary Fig. S9B, C). In PPARα-sufficient mice, AMS treatment overall tended to increase the expression of genes of the innate immunity, in contrast to that of *Flg* and *Aqp3* (Supplementary Fig. S10B). Thus, PPARα deficiency-induced skin dysbiosis triggers oxidative stress associated with AQP3 and SOD2 up-regulation as well as lipid peroxidation and abnormal differentiation in mouse keratinocytes. In contrast, enhanced innate immunity and Th17 in keratinocytes is a direct effect of PPARα deficiency.

## Discussion

Here we uncovered novel functions of PPARα in mouse epidermis, notably a role in reshaping the skin’s bacterial landscape. PPARα deficiency reduced the quantity of bacteria in mouse skin and induced dysbiosis, especially toward an imbalance between *Staphylococcus* and other bacteria. This may result from triggered innate immunity, associated with up-regulation of the NOD2 pathway. Moreover, PPARα deficiency-induced skin bacterial dysbiosis leads to oxidative and mitochondrial stress in mouse epidermis and altered keratinocyte differentiation, with strong effects on FLG.

The skin microbiota is regulated by host innate mechanisms, including low skin-surface pH and temperature, moisturizing factors and antimicrobial lipids and peptides [50–52]. We found that PPARα deficiency decreased the quantity of bacteria in mouse skin and induced skin dysbiosis, mainly in the *Firmicutes* phylum by increasing the proportions of *Streptococcus* and *Bacillus* and reducing those of *Staphylococcus*. *S. aureus* is relatively common in mouse facilities, as demonstrated by publicly available health reports from commercial vendors and is readily transmitted from parents to offspring [53]. Notably, the relative amount of *S. aureus* and *S. lentus* was reduced in the skin of PPARα-deficient mice when compared to littermate controls, in contrast to *S. epidermidis* (Fig. 1). This is in line with previous work showing that PPARα activation worsens superinfection with *S. aureus* in mouse lung via inhibition of the anti-microbial response, including reduced CXCL1 and IL-6 [12].

β-defensins and Th17 cytokines are able to specifically kill specific Staphylococci, with *S. aureus* being the best-studied [54–60]. In line with this, we found up-regulation of *mBD3*, *mBD4* and *Il17c* in keratinocytes and of IL-17A in DETCs of PPARα-deficient mice when compared to littermate controls. Absence of any detectable epidermal barrier defect and of skin infiltration by innate immune cells in PPARα-deficient mouse skin (Supplementary Figs. S1, S4) confirms a preponderant role of antimicrobial peptides and local Th17 immunity in the diminution of *Staphylococcus* colonization [30, 31]. Data from PPARα-sufficient and -deficient mice treated or not with AMS confirm a major role of PPARα in controlling NOD2 and the NOD2 pathway, particularly β-defensins [56, 61, 62]. NOD2 has been shown to induce autophagy [63]. Thus, the presence of autophagosomes (Fig. 2F) only in the epidermis of PPARα-deficient mice further confirms activation of the NOD2 pathway in PPARα-deficient mouse keratinocytes.

Bacterial dysbiosis in the epidermis induces oxidative stress by altering mitochondrial function [43]. The use of topical AMS in PPARα-deficient mice highlighted the role of PPARα-induced skin dysbiosis in oxidative stress and lipid peroxidation in keratinocytes (Figs. 4D-F, 6C). Moreover, we showed that skin bacterial dysbiosis in PPARα-deficient mice affects differentiation processes and notably FLG. Indeed, we found that treatment of PPARα-deficient mouse skin with AMS resulted in a drastic reduction of FLG in mouse epidermis at both the mRNA and protein levels (Fig. 6D). Similar results were found in PPARα-sufficient mice treated with AMS (Supplementary Fig. 10B). Because strong reduction of oxidative stress coincides with FLG down-regulation in PPARα-deficient mice topically treated with AMS, one can hypothesize that skin dysbiosis-driven oxidative stress enhances FLG expression.

In conclusion, these results shed new light on the role of PPARα in epidermis, where PPARα controls skin microbiota composition by triggering skin immunosurveillance via NOD2 activation. This is in line with previous work showing dysbiosis associated with increased innate immunity in the gut of PPARα-deficient mice [13]. However, different mechanisms are operational in mouse intestine versus skin, as the gut dysbiosis was induced by up-regulation of both Th1 and Th17 owing to increased infiltration by macrophages and Il-22-driven up-regulation of antimicrobial peptides [13]. Thus, in skin, PPARα exerts specific functions that are independent of the regulation of lipid and glucose metabolism and which are different from those observed in the gastrointestinal tract.

## Supplementary information

Supplementary material

Supplementary material

## Data Availability

All data are included in the manuscript.
