# Supplementary material for: PPARα deficiency causes skin dysbiosis and triggers innate immunity in keratinocytes
Source: Cell Death Dis. 2026 Apr 14;17(1):479. doi: 10.1038/s41419-026-08640-1 (PMC13183956; doi:10.1038/s41419-026-08640-1)
Supplement: Supplementary file 1 — Supplementary material [file 41419_2026_8640_MOESM1_ESM.pdf]

Supplementary Figure legends:

Supplementary Figure S1: Epidermal barrier in PPARα-deficient mice: TEWL (n=17-18) **(A)** and skin surface pH (n=4-5) **(B)** in PPARα-deficient mice and littermate controls. Data were analyzed with an unpaired Student's t-test.

Supplementary Figure S2: Expression of inflammatory mediators in mouse skin: Relative mRNA levels of *Il17e/ Il25* **(A)**, *Cxcl1* **(B)**, *S100a8* **(C)**, *Vegfa* **(D)**, *Il33* **(E)**, *Il1*a **(F)**, *Tnfa* **(G)** and *Tgfb* **(H)** in mouse epidermis. Data were analyzed with an unpaired Student's t-test, n=8-10. *, $p<0.05$.

Supplementary Figure S3: Immune cells in mouse epidermis: Proportions of cDC1 and cDC2 **(A)**, Langerhans cells **(B)** and DETCs **(C)** in mouse epidermis. **(D)** Gating strategy for DETCs producing IL-17A and IFN-γ. **(E)** Relative mRNA levels of *Mx1* and *Il13* in mouse epidermis. Data were analyzed with an unpaired Student's t-test, n=5-13. *, $p<0.05$; **, $p<0.01$.

Supplementary Figure S4: Reduction of skin bacteria in PPARα-deficient mouse skin does not result from increased recruitment of innate cells to the skin: Proportions of macrophages (M1 and M2) **(A)** and neutrophils **(B)** in mouse epidermis, n=4-10. **(C)** Relative mRNA levels of *Gmcsf* in mouse epidermis, n=4-10. **(D)** Mean fluorescence intensity of CD11b and CD86 at the cell surface of macrophages and neutrophils, n=4-8. **(E)** Numbers of mast cells in mouse skin, n=10. Data were analyzed with an unpaired Student's t-test. *, $p<0.05$; **, $p<0.01$.

Supplementary Figure S5: Markers of DNA damage and mitochondria in mouse epidermis: Representative immunostaining of γH2AX (arrows show positive cells) **(A)** and VDAC **(B)** in mouse epidermis, n=5. Scale bar = 20 μm.

Supplementary Figure S6: AQP3 expression in the epidermis of PPARα-deficient mice: **(A)** Relative mRNA levels of *Aqp3* in mouse epidermis, n=5-10. **(B)** Immunostaining of AQP3

in mouse epidermis, n=5-10. **(C)** Quantity of glycerol in mouse epidermis, n=5-10. Data were analyzed with an unpaired Student's t-test. *, p<0.05.

Supplementary Figure S7: Alterations of epidermal homeostasis in PPARα-deficient epidermis: (A) Representative image of H&E staining of mouse epidermis, n=10. Scale bar = 80 μm. **(B)** Relative mRNA levels of *Spink5* and *Klk5* in mouse epidermis, n=8-10. Data were analyzed with an unpaired Student's t-test.

Supplementary Figure S8: Epidermal lipid metabolism is not affected by PPARα deficiency: mRNA levels of *Echs1*, *Cpt1a*, *Hadhb* and *Acadvl* **(A)**, of *Acox1* and *Hsd17b4* **(B),** and of *Ppard* and *Pparg* **(C)** in mouse epidermis, n=8-10. Data were analyzed with an unpaired Student's t-test.

Supplementary Figure S9: Skin dysbiosis induced by PPARα deficiency alters epidermal homeostasis: mRNA levels of *S100A8* and *Cxcl1* **(A)** and of *Krt1* and *Krt16* **(B)**. **(C)** Numbers of Ki67^{+} cells in PPARα-deficient mouse epidermis treated or not with antimicrobial solution as well as in epidermis of littermate controls, n=8-9. Data were analyzed with an unpaired Student's t-test. *, p<0.05.

Supplementary Figure S10: (A) Quantification of immunostaining expressed as relative mean fluorescence intensity. **(B)** Mice were topically treated with an antimicrobial solution (AMS) as described in Materials and Methods. Relative mRNA levels of *Defb4*, *Il6*, *Il23a*, *Il1b*, *Il17a*, *Nod2*, *Flg* and *Aqp3*. Data were analyzed with an unpaired Student's t-test, n=3-5, *, p<0.05, **, p<0.01.

Supplementary Figure S11: Western blots: Western blots for KRT1, Actin and VDAC (left panel) and for SOD2 (right panel) as described in Materials and Methods.

Supplementary Table S1: Skin cell markers

Supplementary Table S2: Proportions of skin bacterial species

Supplementary Table S3: Material list

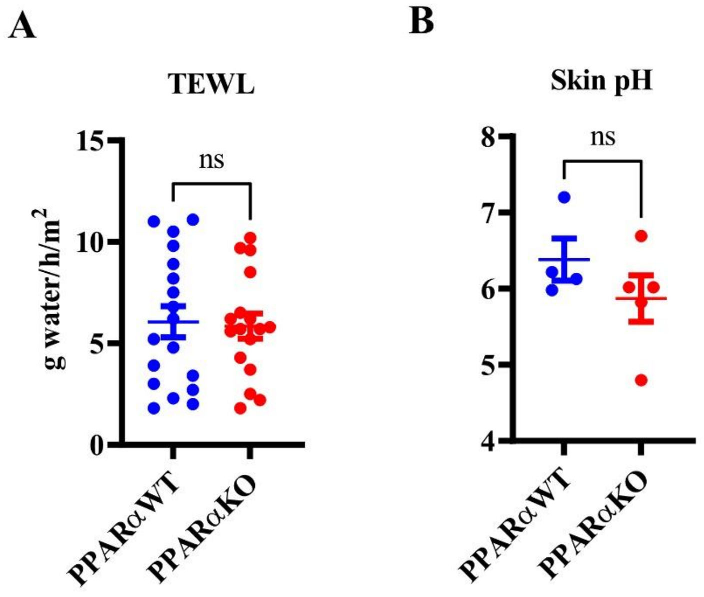
A
B
TEWL
Skin pH
ns
ns
g water/h/m2
15
10
5
0
8
7
6
5
4
PPARαWT
PPARαKO
PPARαWT
PPARαKO

Figure S1

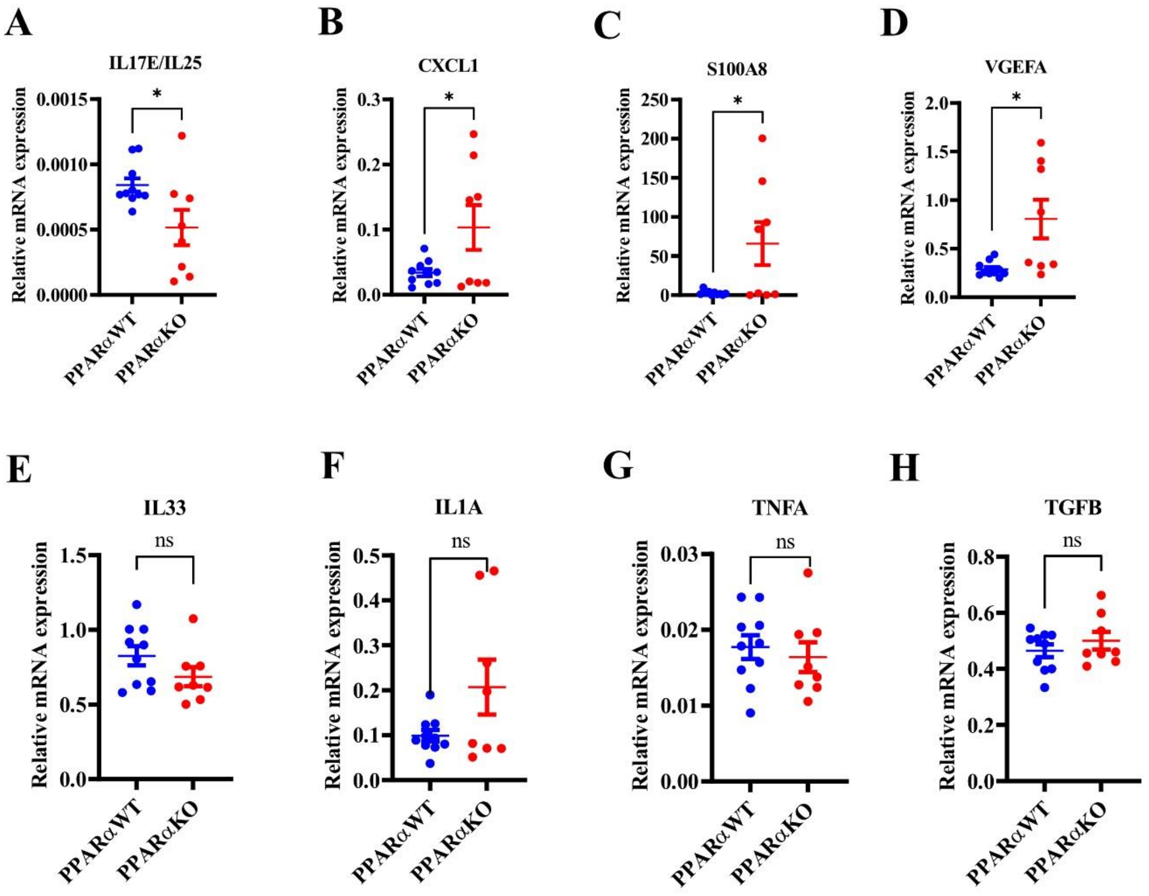
A
IL17E/IL25
*
Relative mRNA expression
0.0015
0.0010
0.0005
0.0000
PPARαWT
PPARαKO
B
CXCL1
*
Relative mRNA expression
0.3
0.2
0.1
0.0
PPARαWT
PPARαKO
C
S100A8
*
Relative mRNA expression
250
200
150
100
50
0
PPARαWT
PPARαKO
D
VGEFA
*
Relative mRNA expression
2.0
1.5
1.0
0.5
0.0
PPARαWT
PPARαKO
E
IL33
ns
Relative mRNA expression
1.5
1.0
0.5
0.0
PPARαWT
PPARαKO
F
IL1A
ns
Relative mRNA expression
0.5
0.4
0.3
0.2
0.1
0.0
PPARαWT
PPARαKO
G
TNFA
ns
Relative mRNA expression
0.03
0.02
0.01
0.00
PPARαWT
PPARαKO
H
TGFB
ns
Relative mRNA expression
0.8
0.6
0.4
0.2
0.0
PPARαWT
PPARαKO

Figure S2

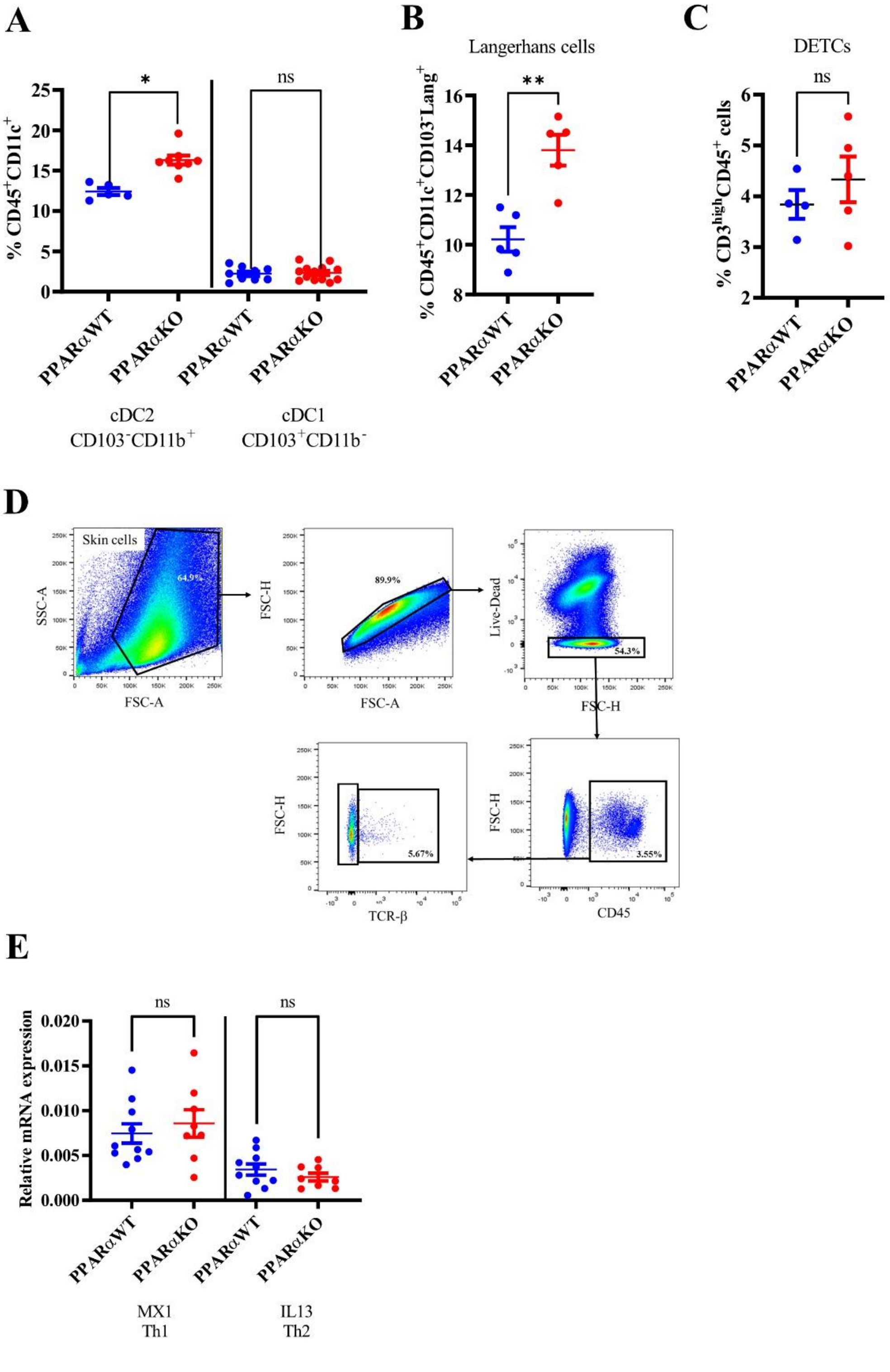
A
*
ns
% CD45+CD11c+
25
20
15
10
5
0
PPARαWT
PPARαKO
PPARαWT
PPARαKO
cDC2
CD103-CD11b+
cDC1
CD103+CD11b-
B
Langerhans cells
**
% CD45+CD11c+CD103-Lang+
16
14
12
10
8
PPARαWT
PPARαKO
C
DETCs
ns
% CD3highCD45+ cells
6
5
4
3
2
PPARαWT
PPARαKO
D
Skin cells
64.9%
SSC-A
FSC-A
89.9%
FSC-H
FSC-A
Live-Dead
54.3%
FSC-H
FSC-H
5.67%
TCR-β
FSC-H
3.55%
CD45
E
ns
ns
Relative mRNA expression
0.020
0.015
0.010
0.005
0.000
PPARαWT
PPARαKO
PPARαWT
PPARαKO
MX1
Th1
IL13
Th2

Figure S3

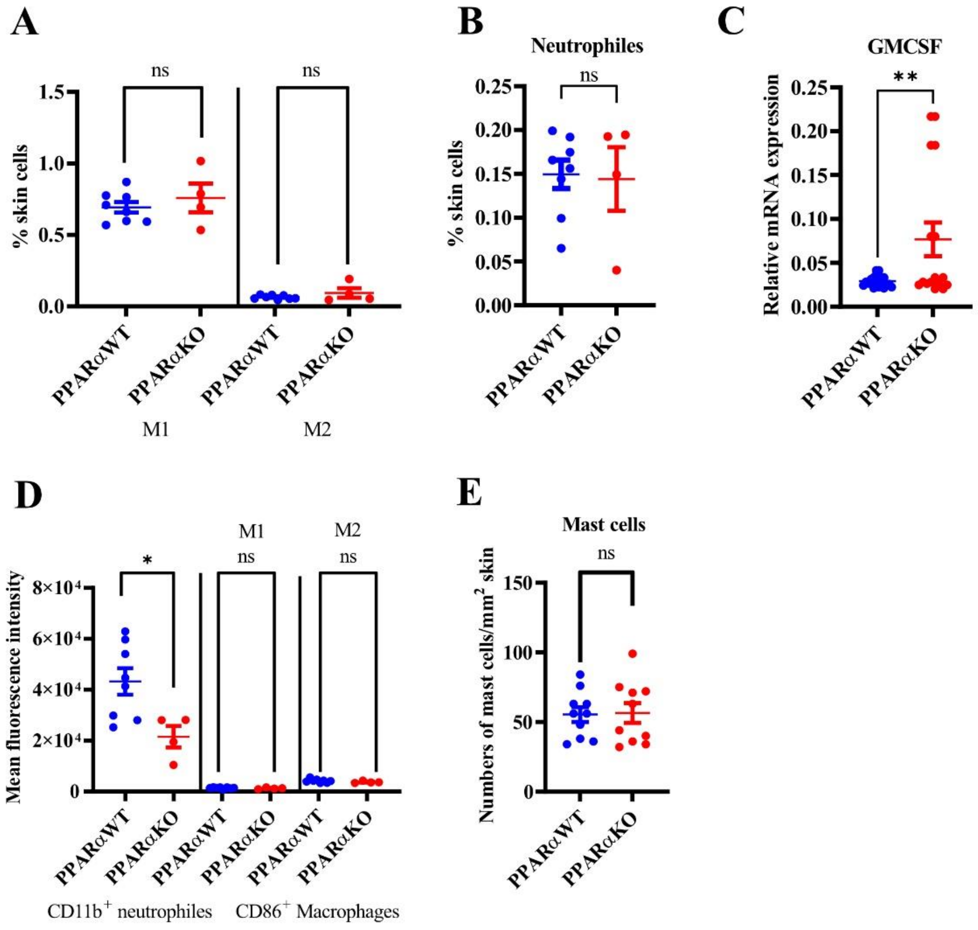

A
ns
ns
1.5
1.0
0.5
0.0
% skin cells
PPARαWT
PPARαKO
PPARαWT
PPARαKO
M1
M2
B
Neutrophiles
ns
0.25
0.20
0.15
0.10
0.05
0.00
% skin cells
PPARαWT
PPARαKO
C
GMCSF
**
0.25
0.20
0.15
0.10
0.05
0.00
Relative mRNA expression
PPARαWT
PPARαKO
D
*
M1
ns
M2
ns
8×10⁴
6×10⁴
4×10⁴
2×10⁴
0
Mean fluorescence intensity
PPARαWT
PPARαKO
PPARαWT
PPARαKO
PPARαWT
PPARαKO
CD11b+ neutrophiles
CD86+ Macrophages
E
Mast cells
ns
150
100
50
0
Numbers of mast cells/mm2 skin
PPARαWT
PPARαKO

Figure S4

A

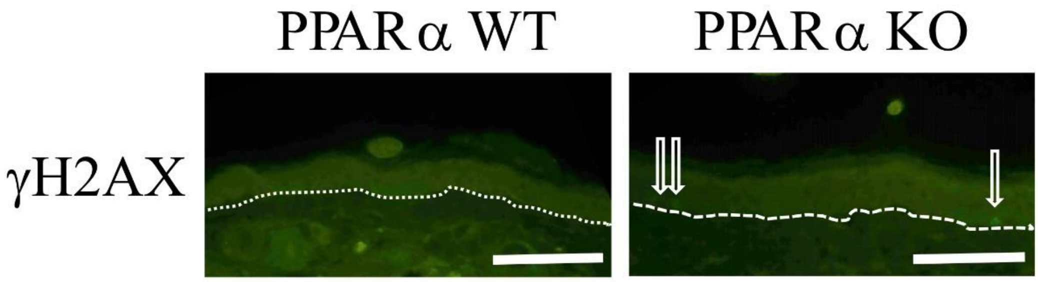

B

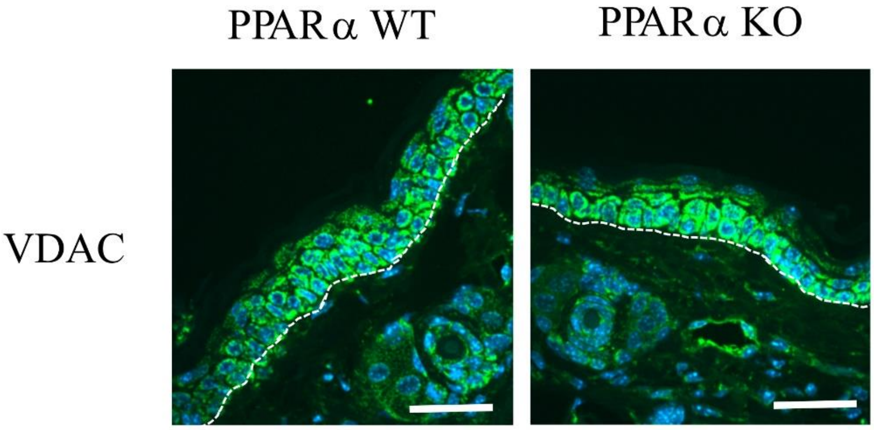

Figure S5

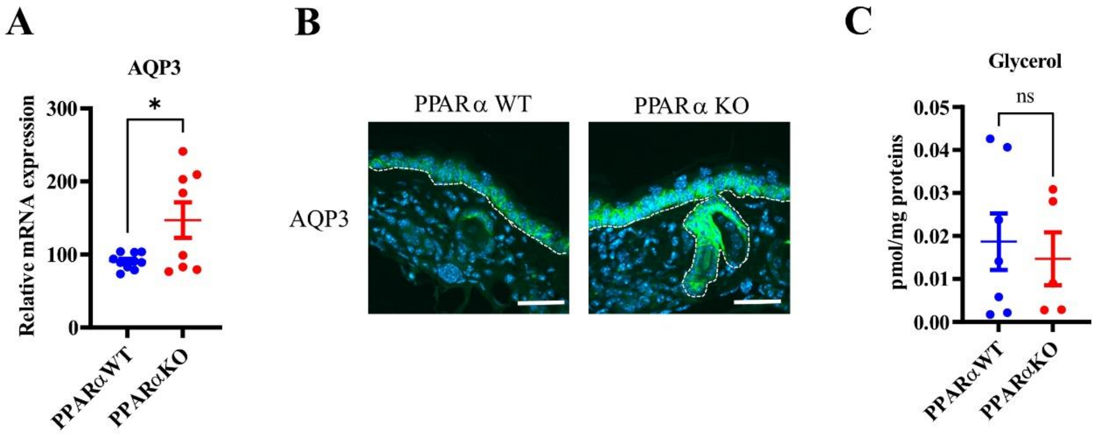
A
AQP3
Relative mRNA expression
300
200
100
0
*
PPARαWT
PPARαKO
B
PPARα WT
PPARα KO
AQP3
C
Glycerol
ns
pmol/mg proteins
0.05
0.04
0.03
0.02
0.01
0.00
PPARαWT
PPARαKO

Figure S6

A

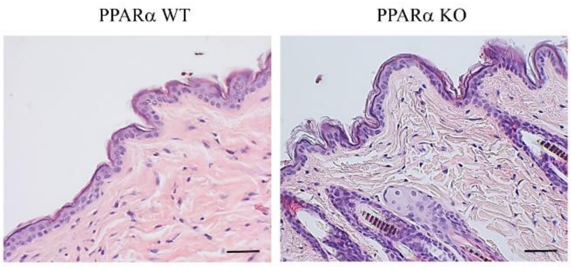
PPARα WT
PPARα KO

B

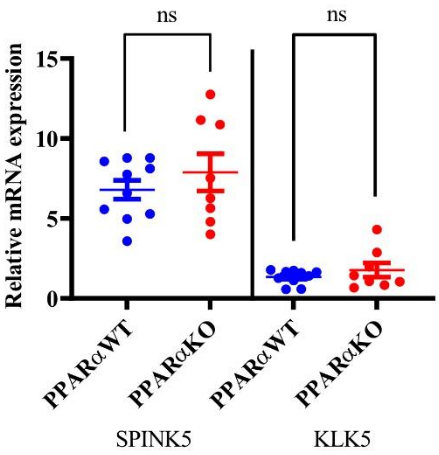
ns
ns
Relative mRNA expression
15
10
5
0
PPARαWT
PPARαKO
PPARαWT
PPARαKO
SPINK5
KLK5

Figure S7

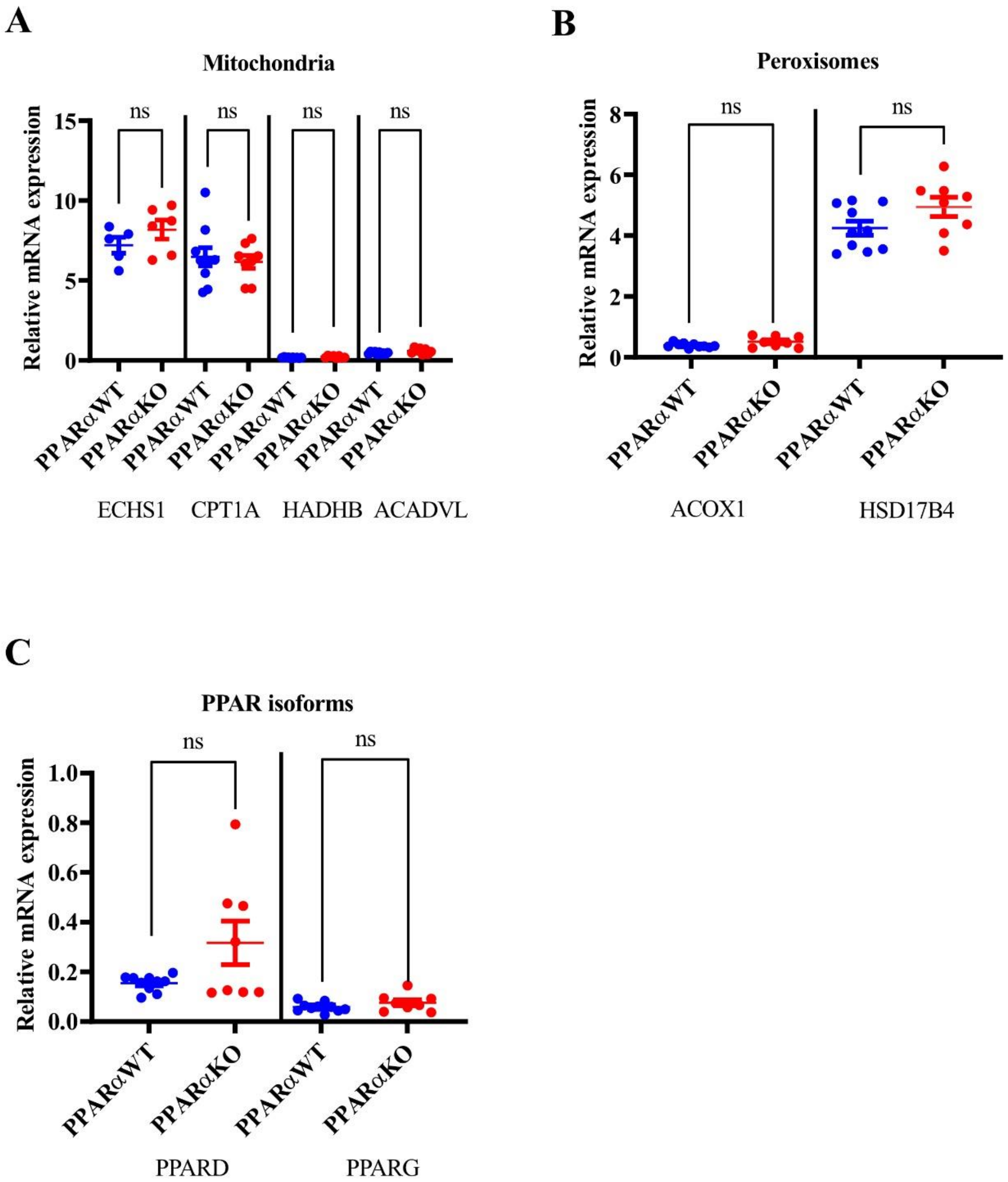
A
Mitochondria
Relative mRNA expression
15
10
5
0
ns
ns
ns
ns
PPARαWT
PPARαKO
PPARαWT
PPARαKO
PPARαWT
PPARαKO
PPARαWT
PPARαKO
ECHS1
CPT1A
HADHB
ACADVL
B
Peroxisomes
Relative mRNA expression
8
6
4
2
0
ns
ns
PPARαWT
PPARαKO
PPARαWT
PPARαKO
ACOX1
HSD17B4
C
PPAR isoforms
Relative mRNA expression
1.0
0.8
0.6
0.4
0.2
0.0
ns
ns
PPARαWT
PPARαKO
PPARαWT
PPARαKO
PPARD
PPARG

Figure S8

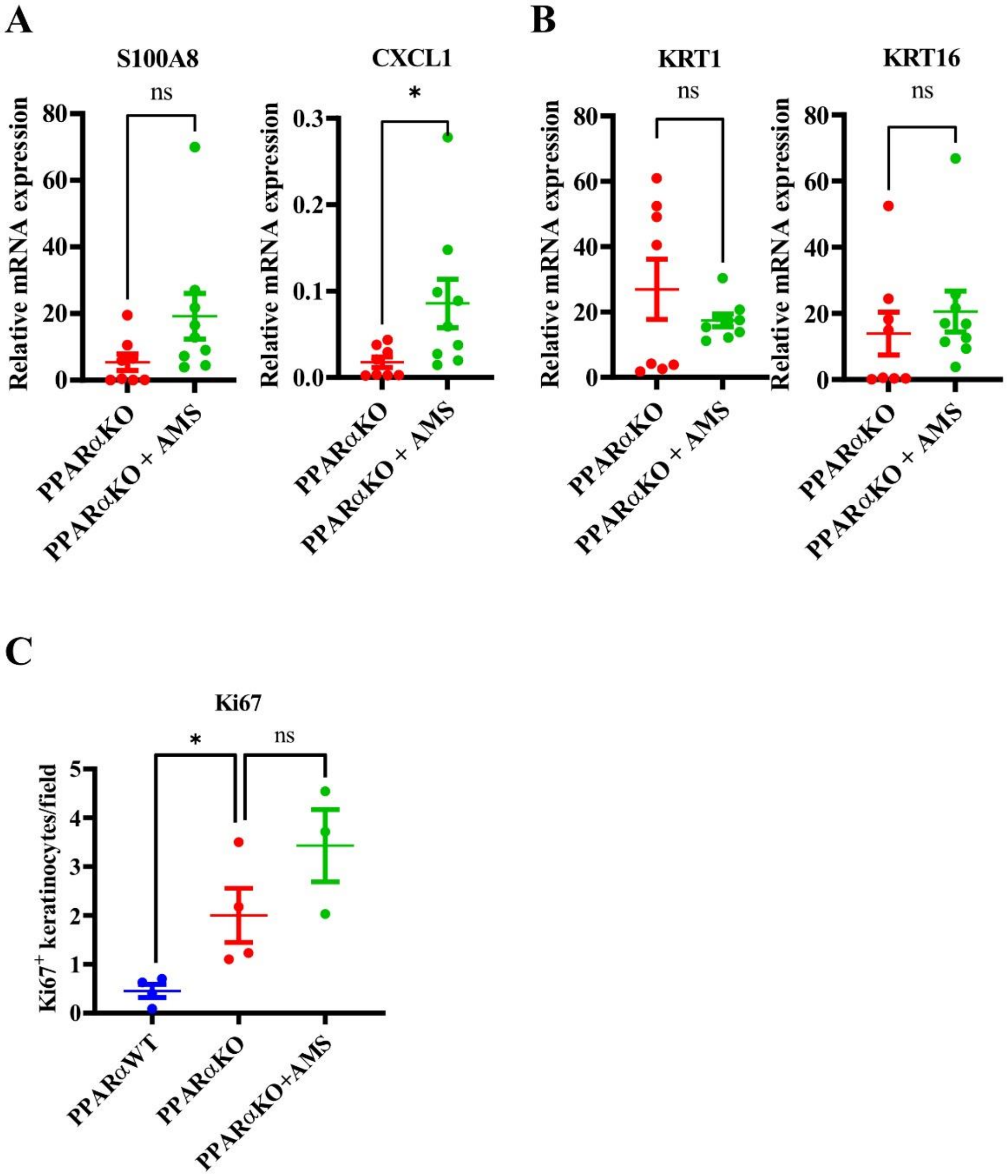
A
S100A8
ns
Relative mRNA expression
80
60
40
20
0
PPARαKO
PPARαKO + AMS
CXCL1
*
0.3
0.2
0.1
0.0
B
KRT1
KRT16
C
Ki67
Ki67⁺ keratinocytes/field
5
4
3
2
1
PPARαWT
PPARαKO+AMS

Figure S9

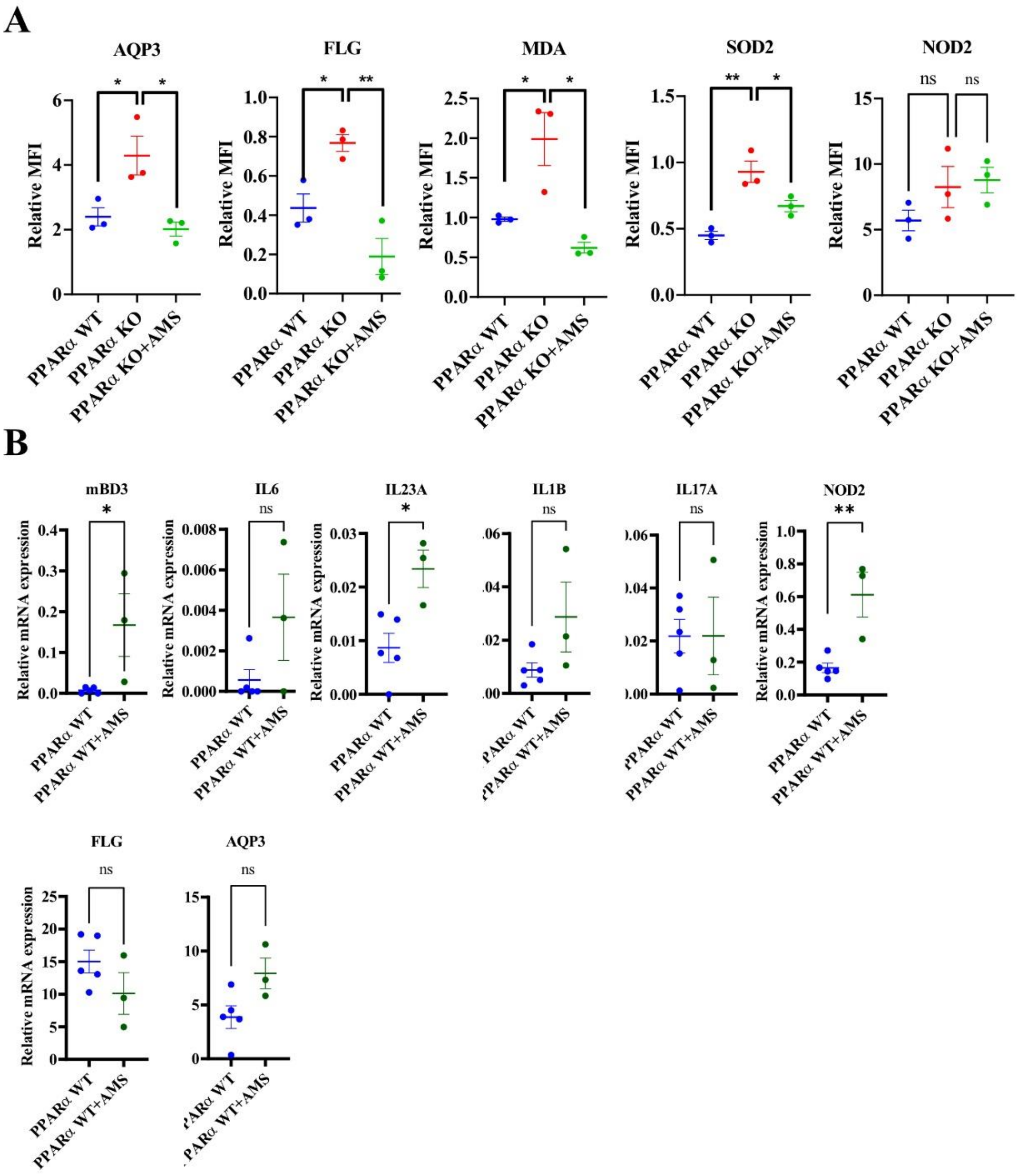
A
AQP3
FLG
MDA
SOD2
NOD2
Relative MFI
PPARα WT
PPARα KO
PPARα KO+AMS
B
mBD3
IL6
IL23A
IL1B
IL17A
NOD2
FLG
AQP3
Relative mRNA expression
PPARα WT
PPARα WT+AMS

Figure S10

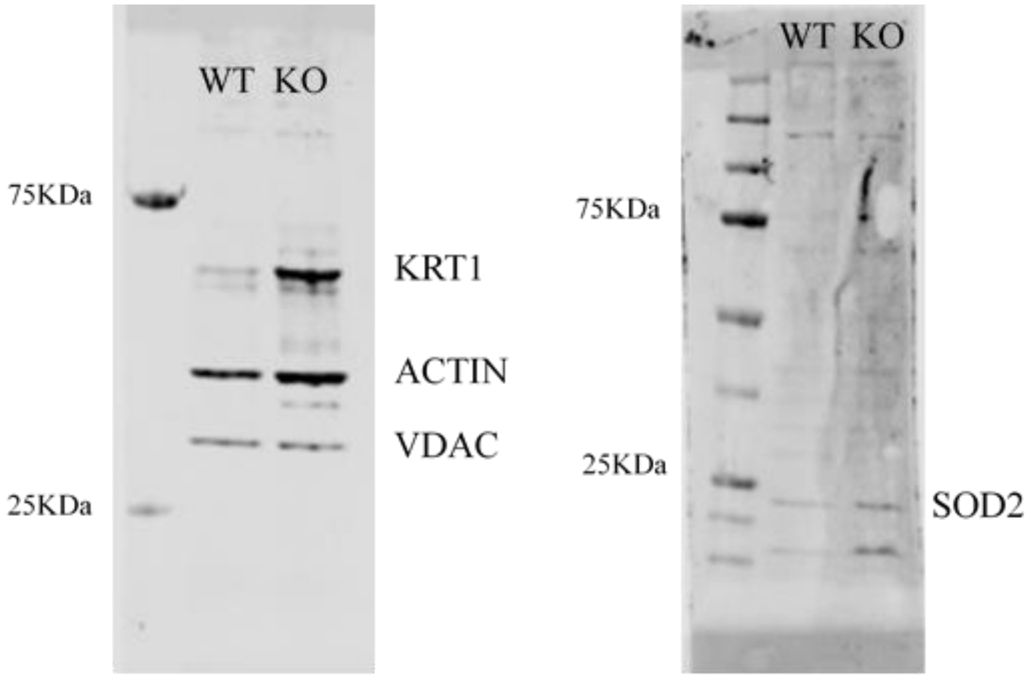

Figure S11

Supplementary Table S1: Skin cell markers

**Cells**	**Identification**
Langerhans cells	$CD45^{+}CD11c^{+}CD103^{-}Langerin^{+}$
Neutrophils	$CD45^{+}CD11b^{+}Ly6G^{high}Ly6C^{+}$
Eosinophils	$Lin^{-}CD45^{+}CD11b^{+}CD193^{+}$ $F4/80^{+}$ $CD86^{+}$
M1 macrophages	$CD45^{+}CD68^{+}CD86^{+}$MHC-class $II^{+}CD163^{-}CLEC7A^{-}$
M2 macrophages	$CD45^{+}CD68^{-}CD86^{+}$MHC-class $II^{+}CD163^{+}CLEC7A^{+}$
DETC	$CD45^{+}CD3^{high}$

Supplementary Table S3: Material list

Reagents and tools table		
**Reagent/Resource**	**Reference of Source**	**Identifier or catalog Number**
**Experimental model**		
PPARα-deficient (M. Musculus)	**(64)**	
**Antibodies for FACS**		
CD3	BD Biosciences	555273
CD11b	BioLegend	79749
CD11c	BD Biosciences	550283
CD103	BD Biosciences	557495
CD163	BioLegend	333621
CD193	BioLegend	144517
CD45	BioLegend	103138
CD68	BioLegend	137013
CD86	BioLegend	105006
CLEC7A	BD Biosciences	749790
F4/80	BioLegend	123108
IFN-γ	BD Biosciences	554413
IL-13	eBioscience	12-7133-81
IL-17A	eBioscience	51-7177-80
Langerin	Dendritics	
Lin	BD Biosciences	558074
Ly6C	BD Biosciences	553104

Ly6G	eBioscience	11-5931-82
MHC class II	557000	557000
**Antibodies for IF and Western blot analysis**		
AQP3	Abcam	ab125219
FLG	Abcam	ab81468
γH2AX	Cell signaling	2577S
Ki67	Roche	05278384001
KRT1	Covance	PRB-149P
mBD3	Santa Cruz	sc-166319
MDA	Abcam	ab6463
NOD2	Invitrogen	PA5-104317
SOD2	Abcam	ab13533
VDAC	ab14734	ab14734
**List of Taqman primers**		
Acadvl	ThermoFischer	Mm00444293_m1
Acox1	ThermoFischer	Mm01246831_m1
Aqp3	ThermoFischer	Mm01208559_m1
Aqp9	ThermoFischer	Mm00508097_m1
Camp	ThermoFischer	Mm00438285_m1
Cpt1a	ThermoFischer	Mm01231183_m1
Cxcl1	ThermoFischer	Mm04207460_m1
Defb3	ThermoFischer	Mm04214158_s1
Defb4	ThermoFischer	Mm00731768_m1
Echs1	ThermoFischer	Mm01276347_m1
Flg	ThermoFischer	Mm01716522_m1

Foxp3	ThermoFischer	Mm00475162_m1
H4c1	ThermoFischer	Mm03031915_gH
Hadhb	ThermoFischer	Mm00695255_g1
Hmox1	ThermoFischer	Mm00516005_m1
Hsd17b4	ThermoFischer	Mm00500443_m1
IL10	ThermoFischer	Mm00439615_g1
Il15	ThermoFischer	Mm00434210_m1
IL17a	ThermoFischer	Mm00439619_m1
Il17c	ThermoFischer	Mm00521397_m1
IL18	ThermoFischer	Mm00434225_m1
IL1a	ThermoFischer	Mm00439620_m1
IL1b	ThermoFischer	Mm00434228_m1
IL22	ThermoFischer	Mm01226722_g1
Il23a	ThermoFischer	Mm01160011_g1
IL25	ThermoFischer	Mm00499822_m1
IL33	ThermoFischer	Mm00505403_m1
IL6	ThermoFischer	Mm00446190_m1
Klk5	ThermoFischer	Mm01203811_m1
Krt1	ThermoFischer	Mm00492992_g1
Krt10	ThermoFischer	Mm03009921_m1
Krt16	ThermoFischer	Mm01306670_g1
Lce3	ThermoFischer	Mm04337256_s1
Mx1	ThermoFischer	Mm00487796_m1
Nod2	ThermoFischer	Mm00467543_m1
Nrf2	ThermoFischer	Mm00477784_m1

Ppara	ThermoFischer	Mm00440939_m1
Ppard	ThermoFischer	Mm00803184_m1
Pparg	ThermoFischer	Mm01184322_m1
S100a8	ThermoFischer	Mm00496696_g1
Sod1	ThermoFischer	Mm01344233_g1
Sod2	ThermoFischer	Mm01313000_m1
Spink5	ThermoFischer	Mm00511522_m1
Tgfb1	ThermoFischer	Mm01178820_m1
Tnfa	ThermoFischer	Mm00443258_m1
Vegfa	ThermoFischer	Mm00437306_m1
S. aureus	ThermoFischer	4368606
S. epidermidis	Vivantis	QM2056
16SrRNA	ThermoFischer	Ba04646229
S. lentus	mfd Diagnostics	
**Chemicals, Enzymes and Other Reagents**		
TrizChlor4®	Dechra	
Ultraclean swab DNA Isolation Kit	MoBio Laboratories	
Liberase	Merck	5401054001
DNase	Thermo Fischer	11284932001
Phorbol 12-myristate 13-acetate	Sigma Aldrich	SIGP8139
Ionomycin	Sigma Aldrich	SIGI3909
Fix&Perm™	AnderGrub Bio-Research	
Live-dead	Thermo Fischer	L23101

7AAD	Sigma Aldrich	SML1633
DAPI	Thermo Scientific	62248
Glycerol kit	Sigma-Aldrich	MAK117
SuperScript® IV Reverse Transcriptase	Thermo Fischer Scientific	18091050
ezDNase	Invitrogen	01337555
Brillant III Ultra-fast	Agilent	600880
TaqMan *S. aureus* detection kit	Applied Biosystems	4368606
**Software**		
GraphPad Prism 10	https://www.graphpad.com	
Analysis pipeline	www.mrdnalab.com	
**Other**		
FACSCanto	BD Biosciences	
Nanodrop 2000c	Thermo Scientific	
Confocal microscope	LEICA DMLS Microscope, Wetzlar	
Electron microscope	Zeiss 10A	
CFX96 Real-Time system	BIO-RAD	
SpectraMax ID3	Molecular Devices	
Fragment analyzer	Advanced Analytical	
